# A83 GENDER-RELATED BARRIERS TO ADVANCEMENT ALONG THE GASTROENTEROLOGY CAREER PATHWAY: AN INTERNAL MEDICINE RESIDENT PERSPECTIVE

**DOI:** 10.1093/jcag/gwad061.083

**Published:** 2024-02-14

**Authors:** A Cintosun, C Pattni, N Jawaid, J Lomonaco, N Bollegala

**Affiliations:** University of Toronto, Toronto, ON, Canada; University of Toronto, Toronto, ON, Canada; University of Toronto, Toronto, ON, Canada; University of Toronto, Toronto, ON, Canada; University of Toronto, Toronto, ON, Canada

## Abstract

**Background:**

Women comprise approximately 31% of the Canadian and 19% of the American gastroenterology population. This gender disparity widens throughout training, with relative gender parity in medical school, slightly less female representation in internal medicine, then with the widest gender disparity in gastroenterology training and practice.

**Aims:**

Our aim was to understand the gender-related barriers to advancement along the gastroenterology training pathway specifically among internal medicine residents.

**Methods:**

A 72-question online survey was distributed by email to internal medicine residents from post-graduate years (PGY) 1, 2, and 3 at the University of Toronto. The initial email was followed by two reminder emails over 1 month. Questions pertained to demographic characteristics, social situation, career aspirations, academic pursuits, and career challenges. Data analysis was performed using SAS software. Continuous variables were compared using Wilcoxon (Mann Whitney U test) and categorical variables with chi-squared and Fisher’s exact test.

**Results:**

40 residents completed the survey. 62.5% were female and most were PGY1 or 2 (n=18, 45% for each; PGY3 was n=4, 10%). Women were more likely to be single (44% vs. 27%, p = 0.0423). In terms of scholarly involvement, women more frequently had additional academic pursuits than their male counterparts (92% vs. 60%, p = 0.0195). For training-related challenges, women more often reported different expectations were put on them due to their due to their background, specifically related to gender (70% vs. 33%, p = 0.0356). Women described more challenges with support staff compared to their colleagues (36% vs. 0%, p = 0.0105), and stated that gender was the reason. Women were also more likely to report that their clinical competence was challenged compared to their peers (32% vs. 7%, p = 0.0384). Outside of work, women more often felt a work-life misbalance due to coordinating childcare (64% vs. 27%, p = 0.0085). Regarding career aspirations, men more often endorsed wanting a hospital leadership role (67% vs. 32%, p = 0.0201). They also rated renumeration as a driving motivation for applying to a subspecialty more often (47% vs. 12%, p = 0.0085) and rated their desired future personal income to over $70,000 more than women (p = 0.0402).

**Conclusions:**

This study is the first to examine gender-related barriers to advancement in gastroenterology at the crucial internal medicine resident level. Women more often reported training-related challenges with others’ expectations and judgments of their clinical competence, as well as personal challenges with coordinating childcare. A deeper understanding of these differences may shape support interventions to help women meet their career goals.

**Funding Agencies:**

None

